# Guidelines for Seizure Prophylaxis in Patients with Aneurysmal Subarachnoid Hemorrhage: A Statement for Healthcare Professionals from the Neurocritical Care Society

**DOI:** 10.1007/s12028-026-02614-z

**Published:** 2026-08-04

**Authors:** A. Shaun Rowe, Sahar F. Zafar, Eljim Tesoro, Emily J. Gilmore, Emily L. Johnson, DaiWai Olson, Appaji Rayi, Jamie Ullman, Yuhong Yuan, Jennifer A. Frontera

**Affiliations:** 1 Department of Clinical Pharmacy and Translational Science, University of Tennessee Health Science Center College of Pharmacy, Knoxville, TN USA; 2 Department of Neurology, Massachusetts General Hospital, Harvard Medical School, Boston, MA USA; 3 Department of Pharmacy Practice, Retzky College of Pharmacy, University of Illinois Chicago, Chicago, IL USA; 4 Department of Neurology, Yale School of Medicine, New Haven, CT USA; 5 Department of Neurology, Johns Hopkins School of Medicine, Baltimore, MD USA; 6 Department of Neurology, UT Southwestern, Dallas, TX USA; 7 Department of Neurology, Charleston Area Medical Center, Charleston, WV USA; 8 Department of Neurosurgery, Zucker School of Medicine at Northwell/Hofstra, Hempstead, NY USA; 9 Department of Medicine, London Health Science Centre, London, ON Canada; 10 Department of Medicine, McMaster University, Hamilton, ON Canada; 11 Department of Neurology, New York University Grossman School of Medicine, New York, NY USA

**Keywords:** Aneurysmal subarachnoid hemorrhage, Prophylaxis, Seizure, Antiseizure medication, Levetiracetam, Phenytoin/fosphenytoin

## Abstract

**Background:**

There are limited data to guide antiseizure medication (ASM) prophylaxis in patients with aneurysmal subarachnoid hemorrhage (SAH). This results in practice variation in the use and duration of ASM prophylaxis.

**Methods:**

We conducted a systematic review and meta-analysis of articles assessing ASM prophylaxis in adults with aneurysmal SAH. The population, intervention, comparator, and outcome (PICO) questions were as follows: (1) Should ASM or no ASM be used in patients hospitalized for aneurysmal subarachnoid hemorrhage who have no history of clinical or electrographic seizures? (2) If an ASM is used, should levetiracetam or phenytoin/fosphenytoin be preferentially used? (3) If an ASM is used, should a long (> 3 days) or short (≤ 3 days) duration of prophylaxis be used? The main outcomes were early seizure (≤ 14 days), late seizures (> 14 days), adverse events, mortality, and functional outcomes. We used Grading of Recommendations Assessment, Development, and Evaluation (GRADE) methodology to generate recommendations.

**Results:**

The initial literature search yielded 1988 articles, of which 10 formed the basis of the recommendations: Regarding PICO 1, we did not find a significant difference in outcomes of early seizure, adverse events, mortality, or functional outcomes when comparing ASM to no ASM. In regard to PICO 2, we found fewer early seizures with phenytoin/fosphenytoin and lower risk for neurologic decline with levetiracetam; however, there was low certainty of evidence. There was no significant difference in mortality between the different treatment types. Regarding PICO 3, we found extended use of ASM may be associated with lower seizure risk, but at the same time be associated with higher risk for adverse effects.

**Conclusions:**

Overall, the quality of evidence is low, precluding strong recommendations. We suggest that ASM or no ASM may be used in patients hospitalized with aneurysmal subarachnoid hemorrhage (conditional recommendation, low quality of evidence). If used, we suggest either levetiracetam or phenytoin/fosphenytoin (conditional recommendation, very low quality of evidence) for a short or long duration (conditional recommendation, moderate quality of evidence).

**Supplementary Information:**

The online version contains supplementary material available at https://doi.org/10.1007/s12028-026-02614-z.

## Introduction

The reported incidence of seizures in the acute phase after aneurysmal subarachnoid hemorrhage ranges from 8 to 24% [1–4]. The rising use of continuous electroencephalography has led to increased detection of nonconvulsive seizures and nonconvulsive status epilepticus [1, 5]. Seizures may be divided further into early versus late on the basis of time of onset relative to aneurysm rupture and initial presentation [4]. Clinical characteristics associated with a higher risk of seizures include age, anterior circulation aneurysm rupture, hydrocephalus requiring cerebral spinal fluid diversion, occurrence of pre-hospitalization seizures, higher clinical grade, aneurysm clipping, and presence of a thick clot [4, 6–11]. There are conflicting reports on the association of early seizures with the development of epilepsy [2]. In addition, seizures, especially in those with a higher burden of nonconvulsive electrographic seizures, are associated with worse cognitive and functional outcomes [5].

There is limited evidence to support the prophylactic use of antiseizure medication (ASM), with most evidence coming from observational cohort studies. The previous American Heart Association guidelines on aneurysmal subarachnoid hemorrhage recommended consideration of prophylactic ASM during the post-bleed period, but they recommend against the use of long-term prophylaxis [12]. However, the 2023 American Heart Association guideline for the management of aneurysmal subarachnoid hemorrhage gives a weak recommendation (i.e., benefit is equal to or greater than the risk) for the use of prophylactic ASM in patients with high-seizure-risk features [9]. In addition, the most current aneurysmal subarachnoid hemorrhage guidelines from the Neurocritical Care Society do not make any recommendations on the use of ASM for seizure prophylaxis [13]. Thus, the authors sought to explore three questions regarding ASM prophylaxis in adult aneurysmal subarachnoid hemorrhage patients with no history of clinical or electrographic seizures: (1) Should ASM or no ASM be used for seizure prophylaxis? (2) If an ASM is used, should levetiracetam or phenytoin/fosphenytoin be preferentially used? (3) If an ASM is used, should a long (> 3 days) or short (≤ 3 days) duration of prophylaxis be used? A summary of the final recommendation for each population, intervention, comparator, and outcome (PICO) question can be found in Table 1.

**Table 1:** Summary of recommendations

	Recommendation	Level of recommendation, quality (certainty) of evidence	Justification
PICO 1	Should antiseizure medication versus no antiseizure medication be used in adult patients hospitalized for aneurysmal subarachnoid hemorrhage who have no history of clinical or electrographic seizures?		
Recommendation 1	Given the uncertainty in benefits and harms, we suggest either that prophylactic ASM be used or withheld or no ASM be used in patients with aneurysmal subarachnoid hemorrhage	Conditional recommendation, low quality of evidence	We did not detect any significant positive or negative effect of ASM compared with no ASM on the outcomes of early seizure, adverse events, or functional outcomes
PICO 2	If antiseizure medication is used, should levetiracetam or phenytoin/fosphenytoin be preferentially used for adult patients with aneurysmal subarachnoid hemorrhage and no history of clinical or electrographic seizures?		
Recommendation 2	We suggest either prophylactic levetiracetam or phenytoin/fosphenytoin in adult patients with aneurysmal subarachnoid hemorrhage	Conditional recommendation, very low quality of evidence	There were no significant benefits to either levetiracetam or phenytoin/fosphenytoin use as seizure prophylaxis regarding either adverse events or mortality
PICO 3	If an antiseizure medication is used, should a long (> 7 days) or short (≤ 7 days) duration of prophylaxis be used in adult patients with aneurysmal subarachnoid hemorrhage and no history of clinical or electrographic seizures?		
Recommendation 3	Regarding the duration of antiseizure medications, because of the uncertainty of benefits and harm, we suggest that the decision to use either short- or long-term therapy with ASM as prophylaxis in adult patients with aneurysmal subarachnoid hemorrhage be individualized to each patient	Conditional recommendation, moderate quality of evidence	While there is evidence from nonrandomized control trials for long duration of prophylaxis with either levetiracetam or phenytoin/fosphenytoin, there are also data describing adverse events associated with phenytoin/fosphenytoin that would be limited by a shorter course

## Methods

This guideline was developed in accordance with Grading of Recommendations Assessment, Development and Evaluation (GRADE) methodology [14, 15], and both panel co-chairs (J.A.F., A.S.R.) completed GRADE workshop training [16].

### Panel Composition

The Seizure Prophylaxis Guideline panel was formed in October 2019 and consists of ten members, including physicians, pharmacists, and nurses with subspecialty experience in neurocritical care, seizure management, trauma, and neurosurgery. In addition, a methodologist (Y.Y.) performed statistical analyses. The panel consisted of six women and four men of diverse racial/ethnic backgrounds (Asian, South Asian, White, and Hispanic). We did not include a patient or the public in our panel. We recognize this as a limitation. In accordance with the GRADE framework, we did approach our outcomes from a patient values perspective during the development of our PICO questions. As the formal involvement of patient and public members in professional guidelines is an evolving standard, we will consider this addition with future revisions.

### Disclosure and Management of Potential Conflicts of Interest

All panel members were required to comply with standard conflict of interest and commercial relationship disclosures, including review of any financial, intellectual, or other relationships that may be construed as a possible conflict of interest. The chairs of the Neurocritical Care Society Guideline Committee, which oversees the Seizure Prophylaxis Guideline Panel, were responsible for vetting any potential conflicts of interest. All members of the Seizure Prophylaxis Guideline Panel were determined to be free of conflicts of interest.

### PICO Generation

Three specific questions were addressed for this guideline following the population, intervention, comparison, outcomes (PICO) format [17]. The PICO questions are as follows: (1) Should ASM or no ASM be used in adult patients hospitalized for aneurysmal subarachnoid hemorrhage who have no history of clinical or electrographic seizures? (2) If an ASM is used, should levetiracetam or phenytoin/fosphenytoin be preferentially used for adult patients with aneurysmal subarachnoid hemorrhage and no history of clinical or electrographic seizures? (3) If an ASM is used, should a long (> 3 days) or short (≤ 3 days) duration of prophylaxis be used in adult patients with aneurysmal subarachnoid hemorrhage and no history of clinical or electrographic seizures?

The outcomes categorized as “critical” (indicating the highest level of importance) included early seizure (either clinical or electrographic) occurring within 14 days of subarachnoid hemorrhage or during hospitalization, late seizure (either clinical or electrographic) occurring ≥ 14 days from hemorrhage or after hospitalization, and adverse events associated with ASM use. A 14-day threshold for early versus late seizure was selected because some studies defined early seizure as within 7 days, others within 14 days, and still others as seizure occurring during hospitalization. Because the threshold of 7 or 14 days is not biologically driven, we chose 14 days to be inclusive of most studies [2, 10, 18–22]. Additional outcomes, including mortality, functional [e.g., modified Rankin Scale scores (mRS), Glasgow Outcome Scale] and cognitive outcomes, were rated as “important.”

### Study Population

This guideline pertains to adult patients hospitalized with acute aneurysmal subarachnoid hemorrhage who do not have a prior history of seizure (clinical or electrographic) or ASM use prior to index injury. Additional guidelines for ASM prophylaxis created for hospitalized patients with supratentorial surgery, intraparenchymal hemorrhage (IPH) [23], and traumatic brain injury (TBI) [24] are published separately.

### Inclusion and Exclusion Criteria

Studies could be included if the following criteria were met: the paper addressed prophylactic ASM use and included an adult population (aged > 18 years) hospitalized with aneurysmal subarachnoid hemorrhage and if data were available on the primary outcomes of interest (early seizure, late seizure, adverse events, mortality, functional outcomes, or cognitive outcomes). Articles were excluded if they involved patients with a history of seizure, epilepsy, or ASM use prior to aneurysmal subarachnoid hemorrhage; were not published in English; were nonhuman studies; were case series with fewer than ten patients; evaluated a pediatric population; or did not assess an outcome of interest. We excluded gray literature, including abstracts, conference proceedings, and non-peer-reviewed articles, as well as review articles and meta-analyses.

### Search Strategy

A search of articles was conducted by a study librarian from 1 January 1946 through 10 July 2020 using PubMed, Medline, Embase, Emcare, and Cochrane databases. Additional literature searches were performed by panel members between 10 July 2020 through 24 September 2024 to capture more recently published articles. Search terms included “seizure,” “antiepileptic medication,” “antiseizure medication,” “levetiracetam,” “Keppra,” “brivaracetam,” “Briviact,” “lacosamide,” “Vimpat,” “phenytoin/fosphenytoin,” “Dilantin,” “phenytoin,” “fosphenytoin,” “Cerebyx,” “valproic acid,” “Depakote,” “carbamazepine,” “lamotrigine,” “prophylaxis,” “prevention,” “prophylactic,” “subarachnoid hemorrhage,” “arachnoidal bleeding,” “aneurysmal subarachnoid hemorrhage,” “mortality,” “death,” “functional outcome,” “function,” “modified Rankin,” “Glasgow Outcome score,” “cognition,” “cognitive,” “disability,” “activities of daily living,” “outcome,” “adverse events,” and “side effects.” Reference lists of published articles, review articles, and meta-analyses were also screened to identify additional articles. See Supplemental Table S1 for the full search strategy.

### Study Screening and Data Collection

Two reviewers independently screened each article title and abstract to determine inclusion eligibility. Full text screening was performed in articles that passed the initial level of review. Screening was performed using DistillerSR software (Ottawa, Ontario, Canada), and all conflicts were adjudicated between reviewers prior to study inclusion. Data were extracted into a standardized tool and classified as randomized controlled trials (RCTs) versus nonrandomized studies, which could be observational studies using retrospective, prospective, cross-sectional, or case series design.

### Risk of Bias and Certainty of Evidence Evaluation

Risk of bias was assessed using the Cochrane Risk of Bias 2 (RoB-2) [25] tool for randomized trials and the Risk of Bias Instrument for Nonrandomized Studies-of Interventions (ROBINS-I) tool [26] nonrandomized studies. These tools were selected on the basis of recommendations from GRADE and the types of articles evaluated. Final risk of bias scores were adjudicated by two reviewers. RoB-2 scoring specifically addresses randomization bias, bias related to deviation from intended interventions, bias due to missing outcome data, bias in measurement of outcome, and bias in selection of the reported result. The ROBINS-I assessment accounted for bias in confounding, bias in patient selection, bias in classification of interventions, deviations from intended interventions, bias from missing data, bias in measurement of outcomes, and bias in selection of the reported result.

**Fig. 1:**
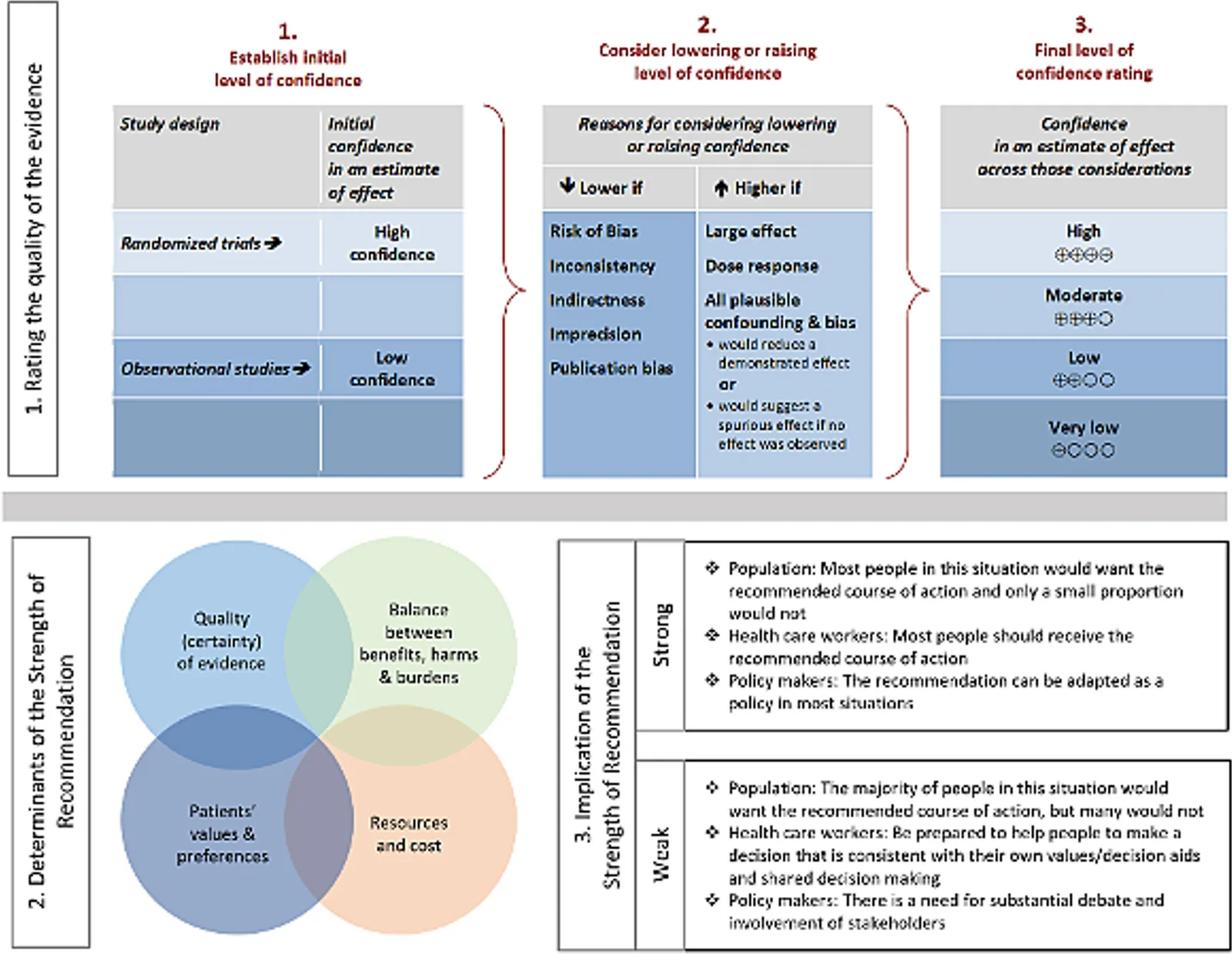
GRADE methodology for rating certainty of evidence and level of confidence and determining the strength of recommendation. Strong recommendations use the term “recommend,” while weak recommendations use the term “suggest.” Unrestricted use of this figure was granted by the US GRADE Network. *GRADE* Grading of Recommendations Assessment, Development, and Evaluation

The certainty of evidence assessment was performed using GRADEPro GDT software (McMaster University and Evidence Prime Inc) according to GRADE methodology [27]. In brief, studies that address a specific outcome of interest can be assessed as a group to determine the certainty with which the evidence leads the panel to make a recommendation. The certainty of evidence may be reduced by the risk of bias, inconsistency (heterogeneity across different studies, typically signified by high *I*^2^ values), indirectness (how closely the studies pertain to the PICO), imprecision [unclear effect size due to low event rates, small sample sizes, or wide confidence intervals (CIs)], and publication bias. The certainty of evidence could be increased by a large effect size, a dose–response gradient, or residual confounding that favors the comparator. A final level of confidence rating is generated from this process ranging from very low to high confidence in the estimate of effect. The certainty of evidence assessment process was conducted and agreed upon by all members of the writing group (Fig. 1).

### Statistical Analyses

All analyses were outcome based and were performed by one study statistician (Y.Y.). For each outcome of interest (early seizure, late seizure, adverse events, functional and cognitive outcomes, mortality), we stratified the analysis by ASM type and by study design (randomized versus nonrandomized studies) and assessed their differences. The summary statistic used for dichotomous data was relative risk (RR), and the mean difference or standardized mean difference was used, when applicable, for continuous data. Studies that reported adjusted odds ratios were pooled using the method of inverse variance. All meta-analyses were conducted using random-effects models. Substantial heterogeneity was defined as *I*^2^ ≥ 50%. All analyses are presented in forest plots and were performed using Revman 5.4 software (Cochrane, London, UK).

### Development of Recommendations

Assessments of judgement for each PICO were performed using GRADEPro GDP software (McMaster University and Evidence Prime Inc). Final recommendations were based on consideration of the importance of the PICO, the certainty and confidence level of the evidence, the balance between the desirable and undesirable effects of the intervention, patient values, and the acceptability and feasibility of the recommendation (Fig. 1). Consensus of all panel members was required for final recommendations. Strong recommendations, which imply that most stakeholders would want to adopt the prescribed guidance and policymakers may use the guideline in most situations, are indicated by the phrase “we recommend.” Conditional recommendations, which imply that most stakeholders would want to adopt the recommendation, though many might not, and that shared decision making between patient and practitioner is likely required, are indicated by the verbiage “we suggest.” The overall quality (certainty) of evidence was averaged across outcomes for each PICO and could be categorized as very low, low, moderate, or high. The limitations in the current body of literature and proposals for future avenues of research are discussed with each PICO.

An “In Our Practice” section is provided in the “Discussion” section, following the formal GRADE-based recommendation and justification. This section highlights current practices [such as dosing, use of electroencephalography (EEG), etc.] that might not be specifically covered in the recommendation. The pragmatic details of this section were determined after panel discussion and represent expert consensus. A caveat to this section is that panel members primarily represent academic centers and reflect current practice in the USA. As such, these suggestions may not be generalizable to all settings.

## Results

A summary of recommendations can be seen in Table 1. The initial literature search yielded 1988 articles, of which 10 formed the basis of the recommendations, and 7 were included in the meta-analyses (Supplemental Fig. 1).

PICO 1: Should antiseizure medication versus no antiseizure medication be used in adult patients hospitalized for aneurysmal subarachnoid hemorrhage who have no history of clinical or electrographic seizures?

**Fig. 2:**
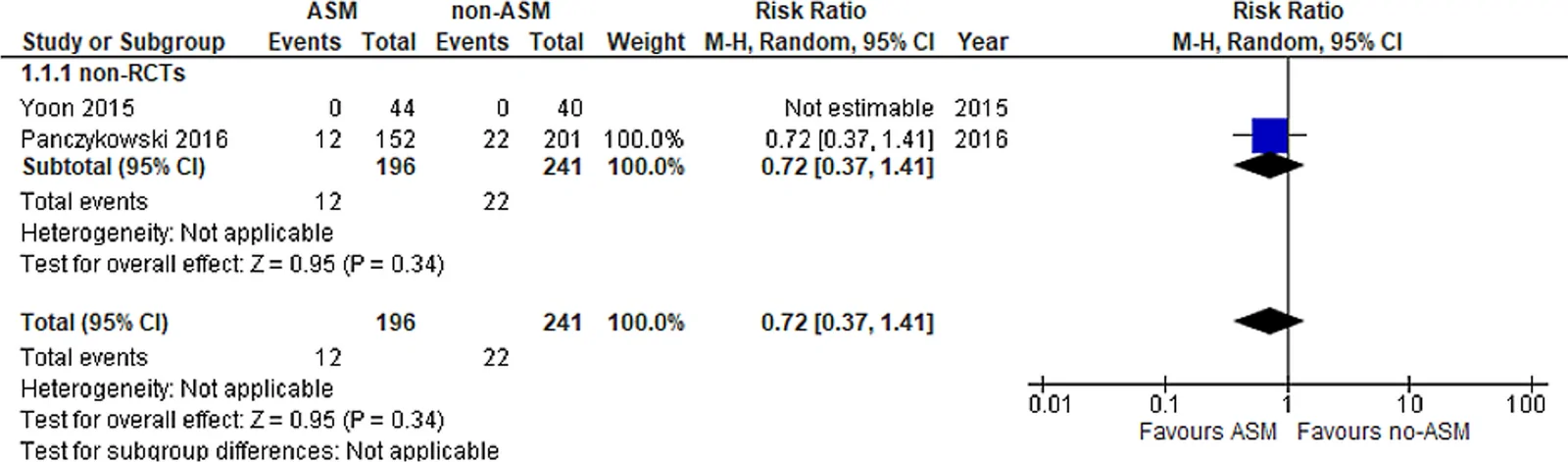
Antiseizure medication versus no antiseizure medication and effect on early seizures after aneurysmal subarachnoid hemorrhage. *M-H* Mantel–Haenszel

**Fig. 3:**
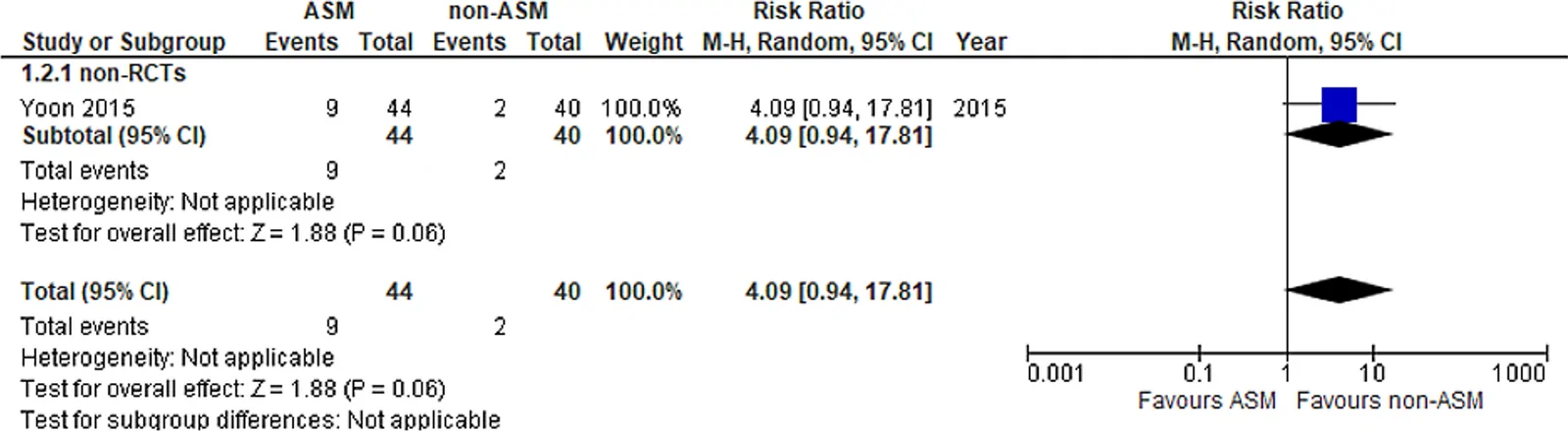
Antiseizure medication versus no antiseizure medication and effect on adverse events after aneurysmal subarachnoid hemorrhage. *M-H* Mantel–Haenszel

**Fig. 4:**
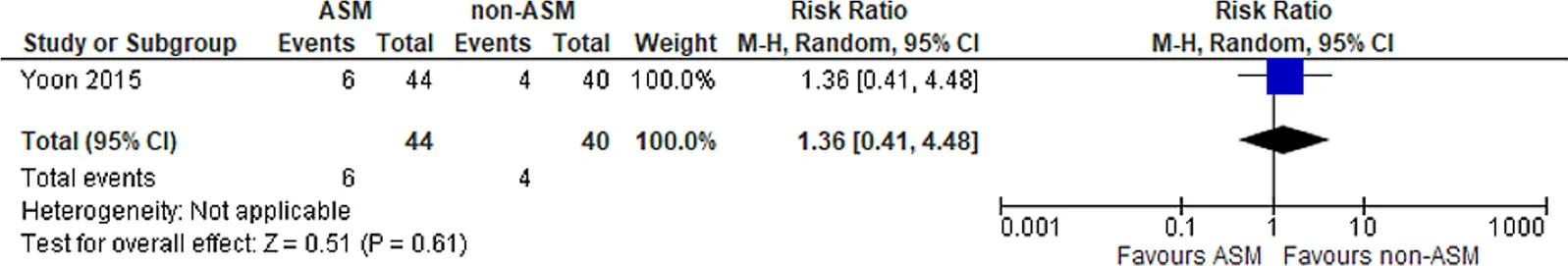
Antiseizure medication versus no antiseizure medication and poor outcomes (mRS 3–6) after aneurysmal subarachnoid hemorrhage. *M-H* Mantel–Haenszel

- To prevent early seizure (≤ 14 days from SAH onset or during hospitalization): Two nonrandomized studies including 437 patients were included in the meta-analysis evaluating the outcome of early seizure [11, 28]. One study compared levetiracetam or valproic acid versus no ASM [28], and the other compared phenytoin/fosphenytoin or levetiracetam versus no ASM [11]. There was no significant reduction in early seizure with the use of ASM versus no ASM (RR 0.72, 95% CI 0.37–1.41; Fig. 2).
- To prevent late seizure (≥ 14 days from aneurysmal subarachnoid hemorrhage onset or after hospitalization): There were no studies examining late seizures in patients treated with ASM versus no ASM.
- Adverse events rates in ASM versus no ASM groups: Only one nonrandomized study (*n* = 84) evaluated adverse events in the ASM versus no ASM group [28]. Adverse events included pneumonia, pulmonary system complications, electrolyte imbalance, cardiac complications, and renal dysfunction. Adverse events occurred in 9/44 in the ASM group versus 2/40 in the no ASM group (RR 4.09, 95% CI 0.94–17.81, *p* = 0.06; Fig. 3).
- Mortality and functional outcomes in ASM versus no ASM groups: One nonrandomized study compared functional outcomes in the ASM (levetiracetam or valproic acid) versus no ASM group (*N* = 84) [28]. Outcomes were examined at discharge and at 6 months postdischarge, and a favorable outcome was defined as an mRS of 0–2 and a poor outcome as an mRS of 3–6. At discharge 6/44 in the ASM group had a poor outcome and 4/40 in the no ASM group had a poor outcome (RR 1.36, 95% CI 0.41–4.48, *p* = 0.61; Fig. 4). At 6 months, there was no change in favorable outcome in the ASM group, while the number of favorable outcomes increased from 36/40 to 38/40 in the no ASM group [28].
- Limitations in the literature: There are several limitations that should be mentioned. First, there were only two studies examining ASM versus no ASM, both of which were nonrandomized and had relatively low sample sizes [11, 28]. One study only included patients with low-grade SAH (Hunt–Hess 1–3), who had an overall lower baseline risk for seizures, poor functional outcomes, and adverse events, which may explain the lack of seizures in this study [28]. The ASM groups were not comparable across the two studies; in one study the ASM group received levetiracetam (1000 mg/day) or valproic acid (900 mg/day) for 6–12 months [28], and in the other the ASM group received levetiracetam (1000 mg every 12 h) or phenytoin/fosphenytoin (100 mg every 8 h) for 7–30 days [11]. In addition, monitoring of drug levels was not reported, particularly for valproic acid and phenytoin/fosphenytoin. Therefore, the prophylaxis regimens are not directly comparable. A standardized definition of early versus late seizures was not used across the studies. One study examined seizures within the hospitalization, along with the time to seizure [11]. Overall, 34/353 patients had clinical or electrographic seizures, with a mean time to seizure onset of 3.6 ± 4.4 days post-ictus, with seizures occurring up to 16 days post-ictus. The majority of patients (16/34) had seizure onset within 24 h of ictus, while 12/34 had seizure onset 1–7 days post-ictus, and 6/34 had seizures 7–16 days post-ictus [11]. No seizures occurred in the ASM or no-ASM group in another study that included patients with only low-grade SAH [28]. The seizure detection method varied across the two studies. One study examined both clinical and electrographic seizures [11]. The other study only examined clinical seizures, and therefore, nonconvulsive seizures may have been missed or not reported [28]. There were very limited data on functional outcomes and adverse effects, with only one study (*n* = 84) examining these outcomes [28].
- Certainty of evidence: The certainty of evidence and effect size for each outcome of interest (early seizure, adverse events, and functional outcomes) are shown (Table 2). For early seizures, adverse events, and functional outcomes the overall certainty of evidence was low. Both studies had very low to moderate risk of bias across all domains.

**Table 2:** Certainty assessment tables for ASM versus no ASM in aneurysmal subarachnoid hemorrhage

Certainty assessment							Number of patients		Effect		Certainty
Number of studies	Study design	Risk of bias	Inconsistency	Indirectness	Imprecision	Other considerations	Antiseizure medication	No antiseizure medication	Relative (95% CI)	Absolute (95% CI)	
**Early Seizure**											
2	Observational studies	Very serious^a,b,c^	Not serious	Not serious	Not serious	All plausible residual confounding would reduce the demonstrated effect	11/196 (5.6%)	9/241 (3.7%)	RR 1.62 (0.69–3.80)	23 more per 1000 (from 12 fewer to 105 more)	⨁⨁⨁◯ Moderate
**Adverse events**											
1	Observational studies	Very serious^a,b,c^	Not serious	Not serious	Serious^c^	All plausible residual confounding would reduce the demonstrated effect	9/44 (20.5%)	2/40 (5.0%)	RR 4.09 (0.94–17.81)	155 more per 1000 (from 3 fewer to 841 more)	⨁⨁◯◯ Low
**MRS 3–6**											
1	Observational studies	Very serious^b,c^	Not serious	Not serious	Not serious	All plausible residual confounding would reduce the demonstrated effect	6/44 (13.6%)	6/40 (15.0%)	RR 1.36 (0.41–4.48)	36 fewer per 1000 (from 162 fewer to 108 more)	⨁◯◯◯ Very low

^a^ Lack of consistent use of clinical and electrographic detection of seizures

^b^ Lack of consistent medication level monitoring

^c^ Single study

Thus, we provide the following recommendation:

Given the uncertainty in benefits and harms, we suggest that prophylactic ASM be used or withheld in patients with aneurysmal subarachnoid hemorrhage (conditional recommendation, low quality of evidence) (Table 3).

**Table 3:** PICO 1 summary of judgements for conditional recommendation for either ASM or no ASM

	Judgement						
Problem	No	Probably no	Probably yes	**Yes**		Varies	Do not know
Desirable effects	**Trivial**	Small	Moderate	Large		Varies	Do not know
Undesirable effects	Large	Moderate	**Small**	Trivial		Varies	Do not know
Certainty of evidence	Very low	**Low**	Moderate	High			No included studies
Values	Important uncertainty or variability	**Possibly important uncertainty or variability**	Probably no important uncertainty or variability	No important uncertainty or variability			
Balance of effects	Favors the comparison	Probably favors the comparison	**Does not favor either the intervention or the comparison**	Probably favors the intervention	Favors the intervention	Varies	Do not know
Acceptability	No	Probably no	**Probably yes**	Yes		Varies	Do not know
Feasibility	No	Probably no	**Probably yes**	Yes		Varies	Do not know

The bold type designates the committee’s judgment

Expert guidance includes the following: In clinical practice, ASM prophylaxis may be considered for patients at high risk of early seizures, including those with high-grade SAH, anterior circulation aneurysms, intraparenchymal hemorrhage, or hydrocephalus requiring cerebral spinal fluid (CSF) diversion, or those undergoing aneurysm clipping. EEG findings may also guide prophylaxis decisions. (See the justification and “In Our Practice” section for details).

Our justification is the following: Across all outcomes of interest, no significant positive or negative effect of ASM compared with no ASM for early seizure, adverse events, or functional outcomes was detected. However, the data were limited and based on small observational nonrandomized studies with confounding. Larger randomized controlled trials that include standardized ASM prophylaxis regimens, standardized definitions of early versus late seizures, and both electrographic and clinical seizure outcomes, along with functional and cognitive outcomes, are indicated. Study drugs should be titrated to therapeutic levels to avoid under-dosing, which can minimize effect size. Subgroup analyses in patients with low- versus high-grade SAH should also be performed. Systematic prospective assessment of adverse events under the oversight of data safety monitoring boards (DSMBs) should also be considered.

PICO 2: If an antiseizure medication is used, should levetiracetam or phenytoin/fosphenytoin be preferentially used for adult patients with aneurysmal subarachnoid hemorrhage and no history of clinical or electrographic seizures?

**Fig. 5:**
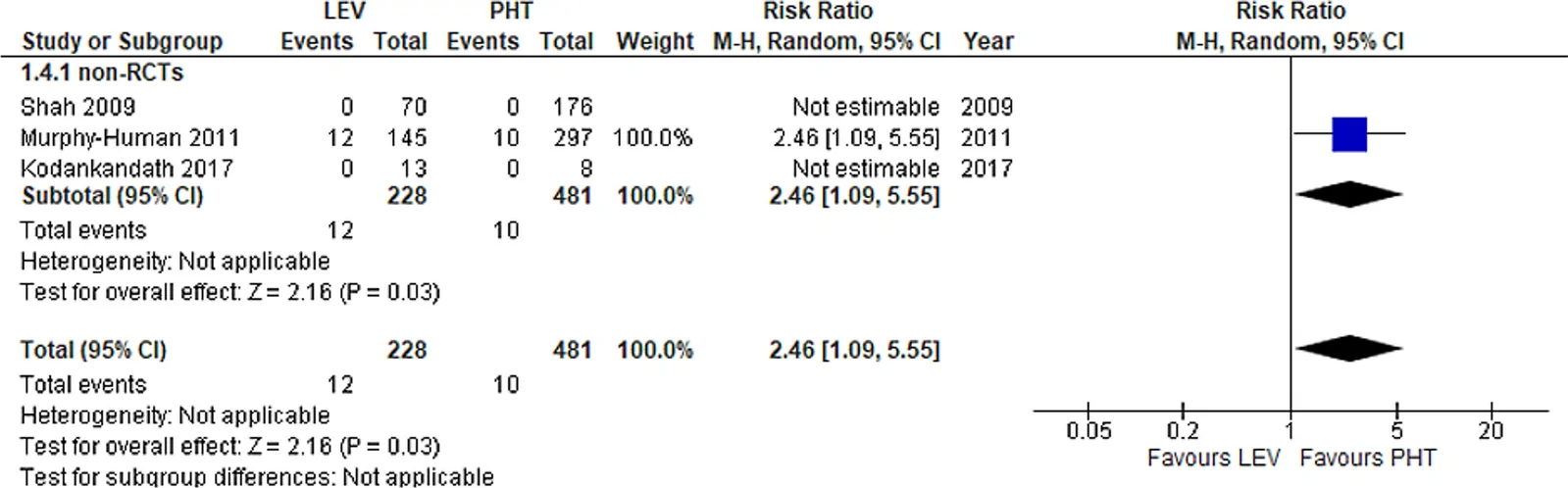
Levetiracetam versus phenytoin/fosphenytoin for the prevention of early seizures in patients with aneurysmal subarachnoid hemorrhage. *M-H* Mantel–Haenszel

**Fig. 6:**
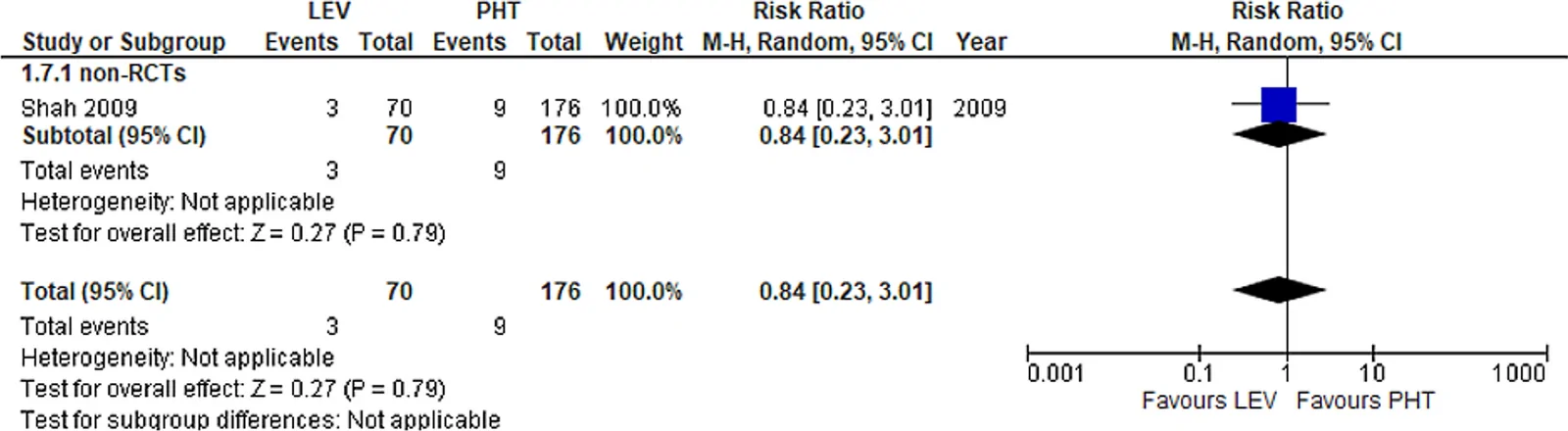
Levetiracetam versus phenytoin/fosphenytoin and effect on neurological outcomes in patients with aneurysmal subarachnoid hemorrhage. *M-H* Mantel–Haenszel

- To prevent early seizure: There were three nonrandomized studies evaluating the effects of levetiracetam versus phenytoin/fosphenytoin in the prevention of early seizures (≤ 14 days), but two of these reported zero events (Fig. 5). One study found early seizures occurred in 12/145 patients on levetiracetam versus 10/297 in patients on phenytoin/fosphenytoin [29]. Overall, a significant difference was found in favor of phenytoin/fosphenytoin prophylaxis (RR 2.46, 95% CI 1.09–5.55), which was driven by data from the non-RCT (Fig. 5) [6].
- To prevent late seizures: No studies comparing levetiracetam and phenytoin/fosphenytoin were found evaluating late seizures.
- Adverse event rates and neurological outcome in levetiracetam versus phenytoin/fosphenytoin: Two non-RCT trials evaluated the effect of levetiracetam or phenytoin/fosphenytoin on adverse events. The first by Kodankandath et al. included 49 patients but reported no adverse events [30]. However, another non-RCT by Shah et al. evaluated the effects of levetiracetam and phenytoin/fosphenytoin on adverse events and found a higher proportion of patients treated with phenytoin/fosphenytoin as compared with levetiracetam experienced elevated transaminases (30.1% versus 0%; *p* < 0.001), thrombocytopenia (11.5% versus 0%; *p* < 0.001), and unexplained fever (5.7% versus 0%; *p* < 0.05). This study also reported data on worsening neurological outcome, which was found to be significantly different in the RCT subgroup favoring levetiracetam (RR 0.35, 95% CI 0.15–0.83) but with a very small sample size. Overall, there was no difference found between levetiracetam and phenytoin/fosphenytoin in terms of worsening neurological outcome (RR 0.84; CI 0.23–3.01) (Fig. 6) [31]. One case–control study (*N* = 259) examined the effects of levetiracetam (including patients switched from phenytoin/fosphenytoin to levetiracetam) or phenytoin/fosphenytoin on neurological outcomes [32]. There was a high rate of switching from phenytoin/fosphenytoin to levetiracetam suggesting poor tolerability [32]. Phenytoin/fosphenytoin was found to be associated more with an mRS > 3 (RR 1.78, 95% CI 1.19–2.66) and the development of delayed cerebral ischemia (RR 1.77, 95% CI 1.35–2.33). However, we excluded this study from our meta-analysis, as many patients experiencing adverse events from phenytoin/fosphenytoin were transitioned to levetiracetam, resulting in medication overlap and confounding of outcomes.
- Mortality in levetiracetam versus phenytoin/fosphenytoin: One non-RCT [29] reported outcomes on mortality. There were no statistical differences seen between levetiracetam and phenytoin/fosphenytoin overall (RR 0.90, 95% CI 0.51–1.61) and in each of the subgroups.
- Limitations: While some studies performed therapeutic serum monitoring to optimize phenytoin/fosphenytoin dosing, levetiracetam was not titrated in a similar fashion from recommended starting doses, raising the possibility of underdosing. Additionally, levetiracetam clearance can be increased in aneurysmal subarachnoid hemorrhage patients due to augmented renal clearance [33], which can result in a potential for breakthrough seizures. The duration of treatment of the two drugs was not consistent across the studies.
- Certainty of evidence: The certainty of evidence was very low to low for all outcomes listed, with no RCTs evaluating levetiracetam and phenytoin/fosphenytoin. Two non-RCT studies reported nonevents for seizure event outcomes leaving only one non-RCT contributing data to this outcome. Risk of bias was deemed to be serious due to retrospective data, lack of trials, and wide confidence intervals (Table 4).

**Table 4:** Certainty assessment tables for levetiracetam vs. phenytoin/fosphenytoin in aneurysmal subarachnoid hemorrhage

Certainty assessment							Number of patients		Effect		Certainty
Number of studies	Study design	Risk of bias	Inconsistency	Indirectness	Imprecision	Other considerations	Levetiracetam	Phenytoin/fosphenytoin	Relative (95% CI)	Absolute (95% CI)	
**Early seizure**											
3	Observational studies	Serious^a^	Not serious	Not serious	Not serious	All plausible residual confounding would reduce the demonstrated effect	12/236 (5.1%)	10/519 (1.9%)	RR 2.46 (1.09–5.55)	27 more per 1000 (from 2 to 82 more)	⨁⨁◯◯ Low
**Gastrointestinal adverse events**											
1	Observational studies	Serious^a,b^	Serious^b^	Not serious	Serious^c^	All plausible residual confounding would reduce the demonstrated effect	1/70 (1.4%)	4/176 (2.3%)	RR 0.63 (0.07–5.53)	8 fewer per 1000 (from 21 fewer to 103 more)	⨁◯◯◯ Very low
**Worse neurological status**											
1	Observational studies	Serious^a,b^	Serious^b^	Not serious	Not serious	None	3/70 (4.3%)	9/176 (5.1%)	RR 0.84 (0.23– 3.01)	8 fewer per 1000 (from 39 fewer to 103 more)	⨁◯◯◯ Very low
**Mortality**											
1	Observational studies	Serious^a,b^	Serious^b^	Not serious	Not serious	All plausible residual confounding would reduce the demonstrated effect	15/145 (10.3%)	34/297 (11.4%)	RR 1.06 (0.21–5.24)	7 more per 1000 (from 90 fewer to 485 more)	⨁◯◯◯ Very low

^a^ Retrospective trials

^b^ Single trial

^c^ Wide confidence interval

Thus, we provide the following recommendation:

We suggest either prophylactic levetiracetam or phenytoin/fosphenytoin in patients with aneurysmal subarachnoid hemorrhage (conditional recommendation, very low quality of evidence) (Table 5).

**Table 5:** PICO 2 summary of judgements for conditional recommendation for either levetiracetam or phenytoin/fosphenytoin in patients with aneurysmal subarachnoid hemorrhage

	Judgement						
Problem	No	Probably no	Probably yes	**Yes**		Varies	Do not know
Desirable effects	**Trivial**	Small	Moderate	Large		Varies	Do not know
Undesirable effects	Large	**Moderate**	Small	Trivial		Varies	Do not know
Certainty of evidence	**Very low**	Low	Moderate	High			No included studies
Values	Important uncertainty or variability	Possibly important uncertainty or variability	**Probably no important uncertainty or variability**	No important uncertainty or variability			
Balance of effects	Favors the comparison	Probably favors the comparison	**Does not favor either the intervention or the comparison**	Probably favors the intervention	Favors the intervention	Varies	Do not know
Acceptability	No	Probably no	Probably yes	**Yes**		Varies	Do not know
Feasibility	No	Probably no	Probably yes	**Yes**		Varies	Do not know

The bold type designates the committee’s judgment

Expert guidance includes the following: Despite limited comparative evidence, levetiracetam is generally preferred due to its favorable safety profile and fewer drug interactions. The observed differences in early seizure prevention could be attributable to appropriate dosing of ASM. Thus, higher dosing may be considered in patients with augmented renal clearance. (See the justification and “In Our Practice” section for details.)

Our justification is the following: There were no significant benefits to either levetiracetam or phenytoin/fosphenytoin use as seizure prophylaxis regarding either adverse events or mortality, as all confidence intervals in the meta-analysis for these outcomes crossed 1.0. In terms of prevention of early seizures, a benefit favoring phenytoin/fosphenytoin was seen in the large non-RCT but was not confirmed in the smaller RCT.

Future studies should evaluate higher levetiracetam doses (or use of loading doses) or use serum level monitoring for titration to therapeutic targets. EEG monitoring should be employed to identify patients with subclinical seizure activity.

PICO 3: If an antiseizure medication is used, should a long (> 3 days) or short (≤ 3 days) duration of prophylaxis be used in adult patients with aneurysmal subarachnoid hemorrhage and no history of clinical or electrographic seizures?

**Fig. 7:**
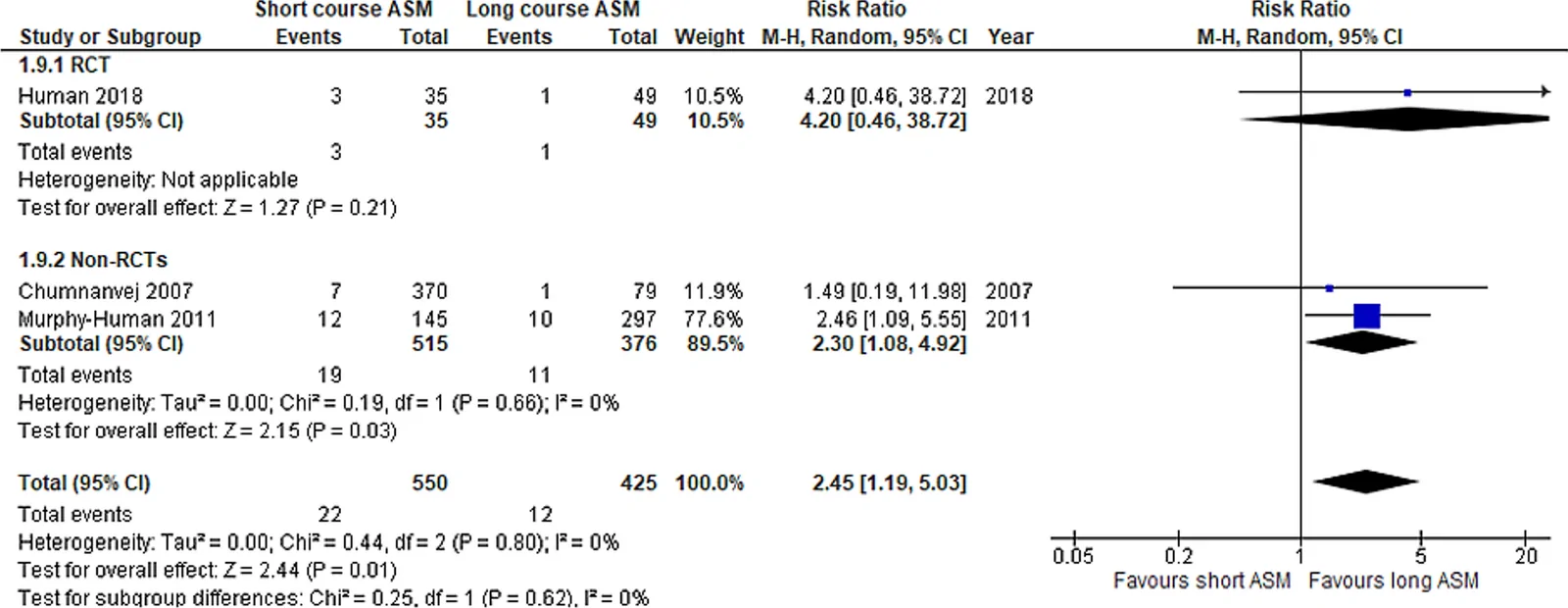
Short-course versus long-course ASM on early seizure in patients with aneurysmal subarachnoid hemorrhage. *M-H* Mantel–Haenszel, *df* degrees of freedom

**Fig. 8:**
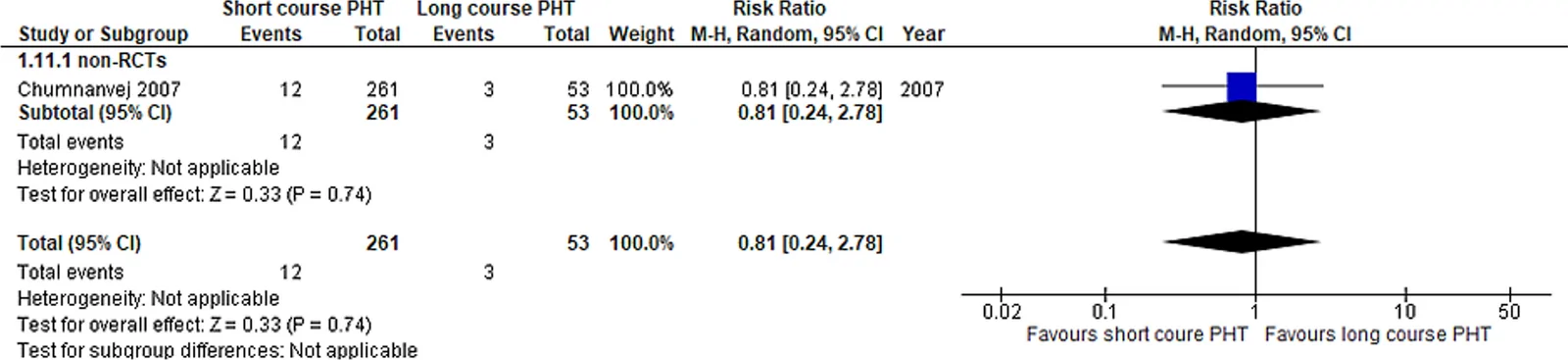
Short- versus long-course effect on late seizure in patients with aneurysmal subarachnoid hemorrhage. *M-H* Mantel–Haenszel

**Fig. 9:**
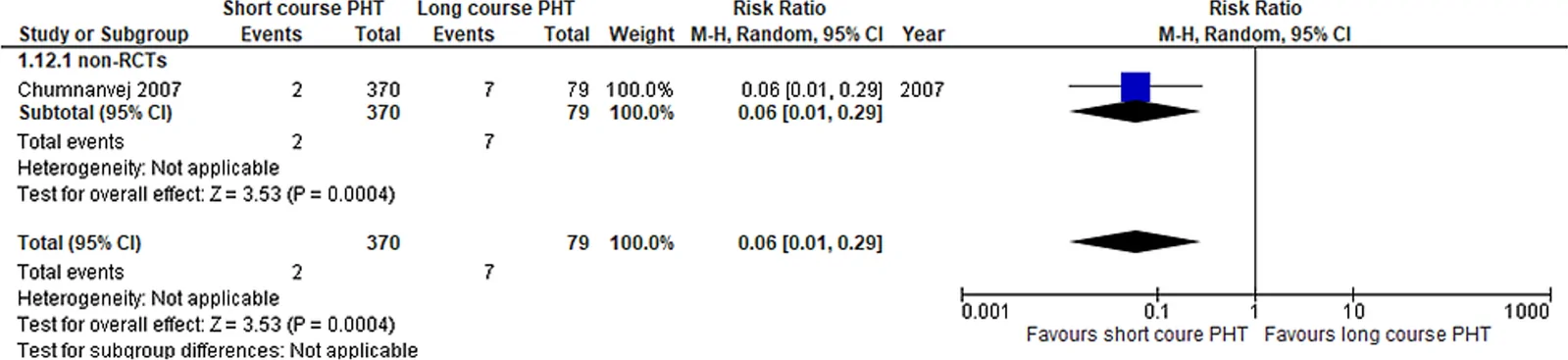
Short- versus long-course effect on adverse events in patients with aneurysmal subarachnoid hemorrhage. *M-H* Mantel–Haenszel

**Fig. 10:**
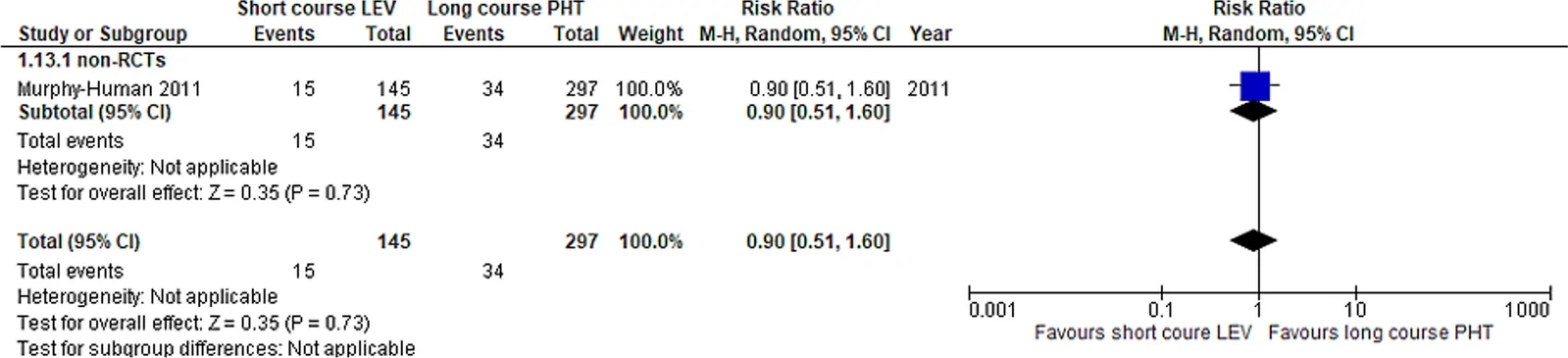
Short- versus long-course effect on mortality in patients with aneurysmal subarachnoid hemorrhage. *M-H* Mantel–Haenszel

**Fig. 11:**
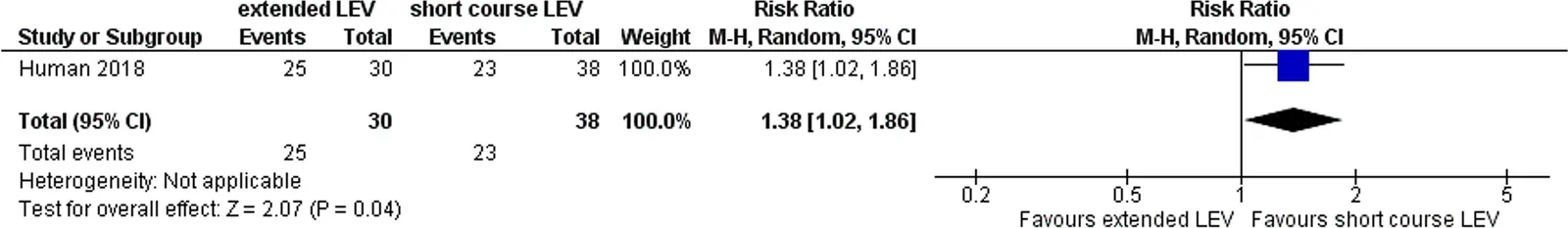
Short- versus long-course effect on good neurological outcomes (mRS 0–2 at follow-up) in patients with aneurysmal subarachnoid hemorrhage. *M-H* Mantel–Haenszel

- Early seizure: Only one prospective, randomized, open-label trial evaluated a short (3 days) versus extended course (until discharge) of levetiracetam prophylaxis in aneurysmal subarachnoid hemorrhage (Fig. 7) [34]. All patients (*N* = 84) received levetiracetam 1000 mg intravenous loading doses and continued 1000 mg twice daily, adjusted for renal function. No differences in in-hospital seizures were reported between the groups (9% versus 2%, *p* = 0.2), but the extended group had more discontinuation of levetiracetam due to sedation (20%). Due to slow enrollment, the study was terminated early and therefore was underpowered. Two non-RCTs were included in the evaluation of early seizure (Fig. 7). The first retrospective study (*N* = 442) compared short-course levetiracetam (≤ 3 days) with extended-course phenytoin/fosphenytoin [29]. In-hospital seizures occurred in more patients on levetiracetam (500 mg twice daily) than phenytoin/fosphenytoin (loaded 15–20 mg/kg followed by a maintenance dose that was titrated to clinical effect or serum levels) (8.3% versus 3.4%, *p* = 0.03), and more late seizures occurred in the levetiracetam group (5.5% versus 2%, *p* = 0.05). It should be noted that the dosing regimen used in this study is low for a population that is known to have augmented renal clearance; thus, this may have affected the outcomes of this study. This may indicate that short-term prophylaxis with levetiracetam may not be effective in preventing seizures and that a longer duration may be necessary. Another retrospective study (*N* = 449) evaluated a 3-day phenytoin/fosphenytoin regimen to a multiweek regimen of phenytoin/fosphenytoin following aneurysmal subarachnoid hemorrhage [35]. The short course of phenytoin/fosphenytoin was associated with reduced hypersensitivity reactions (0.5% versus 8.8%, *p* = 0.002) without an increase in in-hospital seizure frequency (1.9% versus 1.3%, *p* = 0.603) or long-term seizures (4.6% versus 5.7%, *p* = 0.573). Phenytoin/fosphenytoin dosing in this study was a 1000 mg load followed by 100 mg three times daily; no serum drug monitoring was performed. Overall, there was a benefit favoring extended treatment vs. short course (RR 2.45, 95% CI 1.19–5.03), which was driven by data from one non-RCT; the smaller RCT did not support either course of prophylaxis (Fig. 7).
- Late seizures: Only one non-RCT (*N* = 314) looked at the effect of short- versus long-course phenytoin/fosphenytoin to prevent late seizures. One study evaluated phenytoin/fosphenytoin until hospital discharge to a 3-day course, and late seizures were defined on follow-up after discharge, which ranged from 3 to 12 months [35]. Serum levels were not checked in this study. There was no effect of duration of phenytoin/fosphenytoin prophylaxis on prevention of late seizures (RR 0.81, 95% CI 0.24–2.78) (Fig. 8).
- Adverse events: Only one non-RCT comparing a 3-day phenytoin/fosphenytoin course with phenytoin/fosphenytoin until hospital discharge reported all adverse events, which included rash and hepatotoxicity [35]. A significant difference was seen between the two arms favoring a short course of phenytoin/fosphenytoin prophylaxis (RR 0.06, 95% CI 0.01–0.29) (Fig. 9). A similar finding was found looking at phenytoin/fosphenytoin sensitivity using data from the same non-RCT.
- Mortality: Only one non-RCT examined mortality when comparing a brief course of levetiracetam to and extended course of phenytoin/fosphenytoin (*N* = 442). As previously described, no differences in mortality were found with either course (RR 0.9, 95% CI 0.51–1.60) (Fig. 10).
- Neurological outcomes: One small RCT (*N* = 68) evaluated a short course of levetiracetam to an extended course of levetiracetam on the outcome defined as an mRS 0–2 [34]. Patients in the short-course levetiracetam group had a statistically better neurologic outcome (RR 1.38, 95% CI 1.02–1.86) (Fig. 11).
- Certainty of evidence: Both desirable (seizure prevention, mortality) and undesirable (adverse events) effect sizes were deemed small with moderate certainty of evidence overall, as there is very limited data from RCTs (Table 6).

**Table 6:** Certainty assessment tables for short course versus long course ASM in aneurysmal subarachnoid hemorrhage

Certainty assessment							Number of patients		Effect		Certainty
Number of studies	Study design	Risk of bias	Inconsistency	Indirectness	Imprecision	Other considerations	A long duration of antiseizure medication	A short duration of antiseizure medication	Relative (95% CI)	Absolute (95% CI)	
**Early seizure**											
1	Randomized trials	Not serious	Not serious	Not serious	Serious	All plausible residual confounding would reduce the demonstrated effect	1/49 (2.0%)	3/35 (8.6%)	Not estimable	Not estimable	⨁⨁⨁⨁ High
**Early seizure**											
2	Nonrandomized studies	Serious	Serious	Serious	Not serious	All plausible residual confounding would reduce the demonstrated effect	11/376 (2.9%)	19/515 (3.7%)	Not estimable	Not estimable	⨁⨁◯◯ Low
**Late seizure**											
1	Nonrandomized studies	Serious	Not serious	Not serious	Not serious	All plausible residual confounding would reduce the demonstrated effect	3/53 (5.7%)	12/261 (4.6%)	Not estimable	Not estimable	⨁⨁⨁⨁ High
**Adverse events**											
1	Nonrandomized studies	Serious	Not serious	Not serious	Not serious	All plausible residual confounding would reduce the demonstrated effect	7/79 (8.9%)	2/370 (0.5%)	Not estimable	Not estimable	⨁⨁⨁⨁ High
**Mortality**											
1	Nonrandomized studies	Serious	Not serious	Not serious	Not serious	All plausible residual confounding would reduce the demonstrated effect	34/297 (11.4%)	15/145 (10.3%)	Not estimable	Not estimable	⨁⨁⨁⨁ High
**Modified Rankin Score of 0–2 at follow-up**											
1	Randomized trials	Not serious	Not serious	Not serious	Serious	None	23/38 (60.5%)	25/30 (83.3%)	Not estimable	Not estimable	⨁⨁⨁◯ Moderate

*CI* confidence interval

Additional prospective, randomized trials are needed to evaluate a short course to extended course in terms of seizure prevention and clinical/economic outcomes. Prospective studies investigating ASM treatment of electrographic seizures are also indicated. More research is needed to identify potential risk factors to predict the need for prolonged or continued seizure prophylaxis. Additional data on appropriate dosing or titration of antiseizure medications for prophylaxis is needed. Most levetiracetam dosing in these studies was not adjusted for any clinical events and may have been underdosed (only one study reported loading and using a higher maintenance dosing strategy). Studies examining the effects of ASM exposure on neurological and cognitive outcomes should be performed. A study looking at phenytoin/fosphenytoin burden found that increased exposure to phenytoin/fosphenytoin in patients with aneurysmal subarachnoid hemorrhage resulted in poor neurological and cognitive outcomes [36].

Thus, we provide the following recommendation:

Regarding the duration of antiseizure medications, because of the uncertainty of benefits and harm, we suggest that the decision to use either short- or long-term therapy with ASM as prophylaxis in adult patients with aneurysmal subarachnoid hemorrhage be individualized to each patient (conditional recommendation, moderate quality of evidence) (Table 7).

**Table 7:** PICO 3 summary of judgements for conditional recommendation for either long or short duration of treatment with an ASM in patients with aneurysmal subarachnoid hemorrhage

	Judgement						
Problem	No	Probably no	Probably yes	**Yes**		Varies	Do not know
Desirable effects	Trivial	**Small**	Moderate	Large		Varies	Do not know
Undesirable effects	Large	Moderate	**Small**	Trivial		Varies	Do not know
Certainty of evidence	**Very low**	Low	Moderate	High			No included studies
Values	**Important uncertainty or variability**	Possibly important uncertainty or variability	Probably no important uncertainty or variability	No important uncertainty or variability			
Balance of effects	Favors the comparison	Probably favors the comparison	**Does not favor either the intervention or the comparison**	Probably favors the intervention	Favors the intervention	Varies	Do not know
Acceptability	No	**Probably no**	Probably yes	Yes		Varies	Do not know
Feasibility	No	Probably no	**Probably yes**	Yes		Varies	Do not know

The bold type designates the committee’s judgment

Expert guidance includes the following: A short course (≤ 3 days) is generally favored unless EEG or clinical features suggest ongoing seizure risk or high-risk EEG findings. Prophylactic ASM should not be continued beyond 3 days in patients without seizure. (See the justification and “In Our Practice” section for details.)

Our justification is the following: While there is evidence from non-RCTs for long duration of prophylaxis with either levetiracetam or phenytoin/fosphenytoin, there are also data describing adverse events associated with phenytoin/fosphenytoin that would be limited by a shorter course. Data from only one small RCT [34] favors brief course of levetiracetam for neurologic outcome, but this study was terminated early. Larger, well-controlled trials with EEG evaluation, therapeutic drug monitoring, and comprehensive adverse event surveillance are needed.

## Discussion

This “In our practice” section is intended to give practical recommendations that are expert opinion statements from the authors. While the authors were unable to make definitive statements related to the PICO questions addressed in this guideline due to the lack of direct high-quality evidence related to the specific comparisons and outcomes we hoped to explore, we did have significant discussions related to our practice. Below we describe those discussions with the goal of providing clinical guidance.

### PICO 1

There were only two nonrandomized studies examining ASM versus no ASM for early seizures, with one including low-grade SAH and therefore patients at low risk for seizures [4, 5]. The data for adverse effects, mortality, and functional outcomes were similarly limited. While ASMs may be associated with more adverse events, there was no significant difference in overall adverse events or functional outcomes between patients receiving versus those not receiving prophylaxis [4]. There were no studies examining long-term seizure risk. With the current data, many practitioners may use short-term (e.g., until aneurysm is secured, or for up to 3 days) ASM prophylaxis particularly in patients with high risk of early seizures, including those with higher-grade SAH, anterior circulation aneurysms, concomitant intraparenchymal clot, hydrocephalus requiring CSF diversion, or those undergoing clipping versus coiling. An additional consideration is the use of continuous EEG monitoring, when available, to guide ASM prophylaxis. Practitioners may consider ASM prophylaxis if EEG demonstrates high risk patterns, which may include rhythmic and periodic patterns (excluding generalized rhythmic delta) at > 2 Hz, sporadic epileptiform discharges, lateralized periodic discharges, or lateralized rhythmic delta activity; superimposed plus features as defined by the American Clinical Neurophysiology society; and brief ictal rhythmic discharges [37, 38].

### PICO 2

If a prophylactic ASM is used, most practitioners consider levetiracetam over phenytoin/fosphenytoin. While limited data show phenytoin/fosphenytoin may have a better profile at preventing early seizures, this is offset by the higher risk of adverse effects from phenytoin/fosphenytoin. However, strong recommendations are precluded by the low quality of evidence and nonsignificant findings. Additional data reveal phenytoin/fosphenytoin is associated with worse functional outcomes in patients with aneurysmal subarachnoid hemorrhage [36]. This is likely in part due to the potential drug–drug interaction between nimodipine and phenytoin, as phenytoin/fosphenytoin is an inducer of CYP3A4, the enzyme responsible for nimodipine metabolism. As such, phenytoin/fosphenytoin may cause a subtherapeutic level of nimoduipine, which could lead to worse neurological outcomes. Considering these data, if electing to use ASM prophylaxis, practitioners prefer levetiracetam due to better safety profile and tolerability. Levetiracetam has fewer drug–drug interactions, another important consideration for patients with critical illness. Levetiracetam is generally dosed initially at 500 mg two times a day although higher maintenance doses of 750–1000 mg two times a day may be considered, particularly in light of the potential for augmented renal clearance commonly observed in aneurysmal subarachnoid hemorrhage patients [39]. In a study evaluating the population pharmacokinetics of levetiracetam in a heterogeneous sample of patients with transient myocardial ischemia (TMI) and SAH, authors found a high rate of augmented renal clearance necessitating higher doses of levetiracetam to achieve adequate plasma levels [33]. In a heterogenous neurocritical care cohort of TBI, SAH, and IPH, levetiracetam at a dose of 750–1000 mg two times a day resulted in higher odds for achieving target levels and lower odds for seizures [24]. Another study of patients with aneurysmal subarachnoid hemorrhage and TBI found decreased clinical and electroencephalographic seizure activity associated with total daily doses of levetiracetam > 1000 mg [40]. Finally, in a multicenter, prospective, observational study evaluating the optimal dosage of levetiracetam in patients in neurocritical care, the minimum dosing recommended to achieve appropriate plasma levels for patients with augmented renal clearance was 1500 mg twice daily [41].

### PICO 3

If prophylactic ASM is used, we consider using a short course. Thereafter, ASM prophylaxis may be continued using EEG monitoring for guidance. High-risk features on EEG (e.g., high 2HELPS2B score) may be used to guide ASM prophylaxis for a longer duration. We do not favor using ASM prophylaxis beyond 3 days in patients with SAH who do not have clinical or electrographic seizures or high-risk EEG findings. For patients with confirmed clinical or electrographic seizures, we consider continuing ASM treatment at hospital discharge, with close-interval follow-up within 3 months to guide further ASM management.

## Conclusions

A summary of recommendations can be found in Table 1. Overall, the quality of evidence is low, precluding strong recommendations. Majority of the studies are nonrandomized, with variable definitions of early versus later seizures, variable medication regimens, and treatment duration. There are limited data on drug monitoring, adverse events, drug interactions, and use of EEG to guide ASM prophylaxis. Large randomized controlled trials and comparative effectiveness studies are indicated, including studies of EEG-guided ASM treatment. Further data are needed on the long-term impact of treatment, both with regards to cognitive and functional outcomes, and risk for epilepsy development.

## Supplementary Information

Below is the link to the electronic supplementary material.

Supplementary file 1.

Supplementary file 2.

Supplementary file 3.

## Artificial Intelligence

No generative artificial intelligence tools were used in the writing of this manuscript.
