# Supplementary material for: Guidelines for Seizure Prophylaxis in Patients with Aneurysmal Subarachnoid Hemorrhage: A Statement for Healthcare Professionals from the Neurocritical Care Society
Source: Neurocrit Care. 2026 Aug 4;45(2):445–63. doi: 10.1007/s12028-026-02614-z (PMC13642444; doi:10.1007/s12028-026-02614-z)
Supplement: Supplementary file 1 [file 12028_2026_2614_MOESM1_ESM.docx]

**PRISMA 2009 Flow Diagram**

Records excluded
N = 1478

Records excluded:

Not related to PICO questions

Animal or non-human studies

Case series with N<10

Non-English language

Review article

Meta-analysis

Full-text articles excluded, with reasons

N = 390

Full-text articles excluded, with reasons:

Inclusion of patients with prior seizure disorder or ASM use

Not related to PICO questions or outcomes of interest

Wrong population (not non-traumatic ICH)

Records after duplicates removed
N = 1973

Additional records identified through other sources
N = 0

Records identified through database searching
N = 1988

Full-text articles assessed for eligibility
N = 400

Records screened
N = 1973

Studies included in quantitative synthesis (meta-analysis)
N = 7

Studies included in qualitative synthesis
N = 10

## Identification

## Eligibility

## Included

## Screening
