# Supplementary material for: Guidelines for Seizure Prophylaxis in Patients with Aneurysmal Subarachnoid Hemorrhage: A Statement for Healthcare Professionals from the Neurocritical Care Society
Source: Neurocrit Care. 2026 Aug 4;45(2):445–63. doi: 10.1007/s12028-026-02614-z (PMC13642444; doi:10.1007/s12028-026-02614-z)
Supplement: Supplementary file 2 [file 12028_2026_2614_MOESM2_ESM.docx]

**Supplemental Table 1:** Medical Librarian Search Strategy. To 'read' the search strategy - there is a MeSH term (has a '/'), if available followed by synonyms for the MeSH term (has .tw,kf). If there is no MeSH term, then the synonyms come from the relevant EMTREE term in Embase. Search conducted using Ovid MEDLINE(R) ALL 1946 to July 10, 2020

| **#** | **Searches** | **Results** |
| --- | --- | --- |
| 1 | Anticonvulsants/ | 51067 |
| 2 | ("anti convulsant agent?" or "anti epileptic agent?" or "anti epileptic drug?" or "anticonvulsant?" or "anticonvulsive agent?" or "anticonvulsive drug?" or "anticonvulsivum" or "antiepileptic agent?" or "antiepileptic barbiturate?" or "antiepileptic drug?" or antiepileptics or "antiepileptiform drug?").tw,kf. | 44053 |
| 3 | (brivaracetam or briviact or brivlera or nubriveo or rikelta or "ucb 34714" or "ucb34714").tw,kf. | 268 |
| 4 | Cannabidiol/ | 1610 |
| 5 | (cannabidiol or epidiolex or epidyolex or "gwp 42003p" or "gwp42003p" or nabidiolex).tw,kf. | 2861 |
| 6 | Carbamazepine/ | 11105 |
| 7 | (amizepin or amizepine or atretol or biston or calepsin or camapine or carbadac or carbamazepin or carbamazepine or carbategral or carbatol or carbatrol or carbazene or carbazep or carbazepin or carbazina or carmaz or carnexiv or carpaz or carzepin or carzepine or clostedal or epileptol or epimax or epitol or equetro or espa-lepsin or finlepsin or foxalepsin or "g 32883" or "g32883" or hermolepsin or karbamazepin or kodapan or lexin or mazepine or mazetol or neugeron or neurotol or neurotop or nordotol or panitol or servimazepin or sirtal or "spd 417" or "spd417" or tardotol or taver or tegol or tegral or tegretal or Tegretol or tegrital or telesmin or temporol or teril or timonil).tw,kf. | 15445 |
| 8 | (cenobamate or carbamate or "ykp 3089" or "ykp3089").tw,kf. | 7844 |
| 9 | Divalproex/ | 12533 |
| 10 | ("Abbott 50711" or depakote or divalproex or epival or semisodium valproate or valproate semisodium or valprolek).tw,kf. | 924 |
| 11 | Ethosuximide/ | 948 |
| 12 | (asamid or "ci 366" or "ci366" or "cn 10,395" or "cn 10395" or "cn10,395" or "cn10395" or emeside or ethosuccimid or ethosuccimide or ethosuccinimide or ethosuximide or ethylmethylsuccimide or ethylsuximide or ethymal or ethymal or etosuximida or "h 490" or "h490" or mesentol or "nsc 64013" or "nsc64013" or pemal or petimid or petinimid or petinimide or petnidan or "pm 671" or "pm671" or pyknolepsin or pyknolepsinum or ronton or simatin or succinutin or sucsilep or suksilep or suxilep or suximal or suxinutin or zarondan or zarontin).tw,kf. | 1655 |
| 13 | Felbamate/ | 400 |
| 14 | (adipomin or "ahr 965" or "ahr965" or dimafen or fenfluramide or fenfluramin or fenfluramine or fenured or isomeride or kataline or minifage or moderex or obedrex or pesos or phenfluoramine or ponderal or ponderax or ponderex or pondimin or ponflural or rotondin or "val 1177" or "val1177").tw,kf. | 4724 |
| 15 | (fosphenytoin or "acc 9653" or "acc9653" or cerebyx or "ci 982" or "ci982" or fosphenytoin or phosphenytoin sodium or prodilantin).tw,kf. | 492 |
| 16 | Gabapentin/ | 3761 |
| 17 | (apogabapentin or "ci 945" or "ci945" or convalis or dineurin or gabalept or "gabaliquid geriasan" or gabapentin or gabatin or gantin or "go 3450" or "go3450" or "goe 3450" or "goe3450" or gralise or kaptin or keneil or neurontin or neurotonin or nupentin).tw,kf. | 6389 |
| 18 | Lacosamide/ | 498 |
| 19 | ("add 234037" or "add234037" or erlosamide or harkoseride or lacosamide or "spm 927" or "spm927" or vimpat).tw,kf. | 878 |
| 20 | Lamotrigine/ | 3067 |
| 21 | ("bw 430c" or "bw 430c78" or "bw430c" or "bw430c78" or crisomet or labileno or labileno or lambipol or lamepil or lamictal or lamictin or lamiktal or lamitrin or lamodex or lamogine or lamotrigin or lamotrigine or lamotrix or medotrigin or neurium or "seizal (drug)").tw,kf. | 5307 |
| 22 | Levetiracetam/ | 2136 |
| 23 | (elepsia or etiracetam or keppra or kopodex or levetiracetam or "lo 59" or "lo59" or matever or spritam or "ucb 6474" or "ucb l 059" or "ucb l059" or "ucb l060" or "ucb6474").tw,kf. | 3855 |
| 24 | Oxcarbazepine/ | 983 |
| 25 | ("gp 47680" or "gp47680" or "kin 493" or "npc 04" or "npc04" or oxcarbazepine or oxocarbazepine or oxrate or oxtellar or timox or "tri 476" or "tri476" or trileptal or trileptin).tw,kf. | 1972 |
| 26 | Pentobarbital/ | 10156 |
| 27 | (auropan or barpental or diabutal or dorsital or embutal or etaminal or ethaminal or euthanyl or euthesate or isoamytal or iturate or mebubarbital or mebumal or mebumalum or medinox mono or mintal or napental or narcovet or nembutal or palapent or pentabarbitone sodium or pental or pentobarbilake or pentobarbital or pentobarbitalum or pentobarbitone or pentone or pentyl or praecicalm or sagatal or sedalixir or sombutol or somnopentyl or somnotol or sopental or sotyl or vetbutal).tw,kf. | 19353 |
| 28 | Phenobarbital/ | 17953 |
| 29 | (adonal or aephenal or agrypnal or alepsal or amylofene or andral or aparoxal or aphenylbarbit or aphenyletten or atrofen or austrominal or barbapil or barbellen or barbenyl or barbilettae or barbilixir or barbinal or barbiphen or barbiphenyl or barbivis or barbonal or barbonalett or barbophen or bardorm or bartol or bialminal or calmetten or calminal or carbronal or cardenal or cemalonal or codibarbital or coronaletta or cratecil or damoral or dezibarbitur or dormina or dormiral or dromural or ensobarb or ensodorm or epanal or epidorm or epilol or episedal or epsylone or eskabarb or etilfen or euneryl or fenbital or fenemal or fenobarbital or fenolbarbital or fenosed or fenylettae or gardenal or gardenale or gardepanyl or glysoletten or haplopan or haplos or helional or hennoletten or hypnaletten or hypno tablinetten or hypnogen fragner or hypnolone or hypnotal or hypnotalon or hysteps or lefebar or leonal or lephebar or lepinal or lethyl or linasen or liquital or lixophen or lubergal or lubrokal or lumesettes or lumesyn or luminal or luminale or luminaletas or luminalette or luminaletten or luminalettes or luminalum or lumofridetten or luphenil or luramin or menobarb or molinal or neurobarb or nirvonal or noptil or nova pheno or nunol or parkotal or pharmetten or phen bar or phenaemal or phenemal or phenethylbarbital sodium or phenobal or phenobarb or phenobarbital or phenobarbiton or phenobarbitone or phenobarbitural or phenobarbyl or phenonyl or phenotal or phenoturic or phenoyl or phenyl ethyl barbituric acid or phenylbarbital or phenylethyl barbituric acid or phenylethylbarbituric acid or phenylethylmalonyl urea or phenylethylmalonylurea or phenyletten or phenyral or polcominal or promptonal or seda tablinen or sedabar or sedicat or sedizorin or sedlyn or sedofen or sedonal or sedonettes or seneval or sevenal or sombutol mcclung or somnolens or somnoletten or somnosan or somonal or spasepilin or starifen or starilettae or stental or teolaxin or theolaxin or triabarb or tridezibarbitur or "uni-feno" or versomnal or wakobital or zadoletten or zadonal).tw,kf. | 65617 |
| 30 | Phenytoin/ | 13464 |
| 31 | (alepsin or aleviatin or antilepsin or antisacer or cansoin or citrullamon or comital or cumatil or danten or dantoin or denyl or "di hydan" or difenin or difetoin or differenin or difhydan or dihydan or dilantin or dintoin or dintoina or diphantoin or diphantoine or diphedal or diphedan or "di-phen" or diphenin or diphenine or diphentoin or diphenyl hydantoin or diphenylan or diphenyldantoin or diphenylhydantoin or diphenytoin or ditoin or ditomed or dlphenylhydantoin sodium or ekko or epamin or epanutin or epelin or "epilan d" or epilantin or epileptin or eptal or eptoin or felantin or fenantoin or fenitoin or fenytoin or fenytoine or hidanil or hidantal or hydantin or hydantinal or hydantoinal or hydantol or idantoin or lehydan or lepitoin or minetoin or neosidantoina or phenhydan or phenhydane or phenilep or phentytoin or phenybin or phenydan or phenydantin or phenytek or phenytex or phenytoin or phenytoine or phenytoinum or phenytonium or pyoredol or sanepil or sodantoin or sodanton or sodium diphenylhydantoinate or solantoin or solantyl or tacosal or vasilcon or zentropil).tw,kf. | 14977 |
| 32 | Pregabalin/ | 1988 |
| 33 | ("148553-50-8" or "ci 1008" or "ci1008" or lyrica or "pd 144723" or "pd144723" or pregabalin).tw,kf. | 3855 |
| 34 | Primidone/ | 1297 |
| 35 | (cyral or desoxyphenobarbital or desoxyphenobarbitone or hexadiona or lepsiral or liskantin or majsolin or midone or misodine or mizodin or mutigan or mylepsin or mylepsinum or mysolin or mysoline or neurosyn or primaclone or primadone or primidon holsten or primidone or prysoline or resimatil or sertan).tw,kf. | 1269 |
| 36 | Thiopental/ | 6840 |
| 37 | (anesthal or bomathal or farmotal or hypnostan or intraval or leopental or nesdonal or penthiobarbital or penthotal or pentothal or pharmothal or ravonal or "rp 245" or "rp245" or sodipental or sodium pentothal or sodium thiopental or thiomebumal or thionembutal or thionyl or thiopental or thiopentalbarbital or thiopentemal or thiopenthal or thiopentobarbital or thiopentone or thiotal or thiothal or "tio pentemal" or "tiobarbital braun" or "tiopental sodico" or trapanal).tw,kf. | 8541 |
| 38 | Tiagabine/ | 521 |
| 39 | ("nnc 05 0328" or "nnc 328" or "no 05 0328" or "no 05 0329" or "no 328" or "no 329" or gabitril or tiabex or tiagabine).tw,kf. | 939 |
| 40 | Topiramate/ | 2738 |
| 41 | (acomicil or ecuram or epiramat or epitomax or epitoram or erravia or etopro or fagodol or jadix or lusitrax or maritop or "mcn 4853" or "mcn4853" or "mcn-4853" or oritop or piraleps or pirantal or pirepil or qudexy or "ramas (drug)" or "rwj 17021" or "rwj 17021-000" or "rwj17021" or "rwj17021-000" or sincronil or talopam or tiramat or topaben or topamac or topamax or topepsil or topibrain or topilek or topimark or topimax or topiramat or topiramate or topiramato or topiratore or topit or toramat or torlepta or trokendi or "usl255").tw,kf. | 4666 |
| 42 | Trimethadione/ | 490 |
| 43 | (absentol or edion or epidione or mino aleviatin or petidion or ptimal or tridione or trimethadion or trimethadione or trimetin or trioksal or troxidone).tw,kf. | 465 |
| 44 | Valproic Acid/ | 12533 |
| 45 | ("abbott 44090" or absenor or "alpha propylvalerate" or "alpha propylvaleric acid" or apilepsin or atemperator or "convival chrono" or convulex or convulsofin or delepsine or depacon or depakene or depakin or depakine or depakote or depalept or deprakine or diplexil or "dipropyl acetate" or "dipropyl acetic acid" or dipropylacetate or "dipropylacetatic acid" or "dipropylacetic acid" or diprosin or divalproex or epilam or epilex or epilim or episenta or "epival cr" or ergenyl or espa valept or everiden or goilim or hexaquin or "kw 6066 n" or labazene or leptilan or leptilanil or micropakine or mylproin or "myproic acid" or orfil or orfiril or orlept or petilin or "propylisopropylacetic acid" or propymal or stavzor or "valberg pr" or valcote or valepil or valeptol or valerin or "valhel pr" or valoin or valpakine or valparin or valporal or valprax or valpro or valproat or valproate or valprodura or "valproic acid" or valprosid or valprotek or valsup or vupral).tw,kf. | 18173 |
| 46 | Vigabatrin/ | 1587 |
| 47 | (kigabeq or "mdl 71,754" or "mdl 71754" or "rmi 71754" or "rmi 71890" or sabril or sabrilex or vigabatrin or vigadrone).tw,kf. | 1927 |
| 48 | Zonisamide/ | 704 |
| 49 | ("68291-97-4" or "68291-98-5" or "Ad 810" or "ad810" or "ci 912" or "ci912" or excegran or excemid or zonegran or zonisamide).tw,kf. | 1464 |
| 50 | or/1-49 [Antiseizure Antiepileptic Drugs] | 220784 |
| 51 | Seizures/pc | 3856 |
| 52 | exp epilepsy/pc | 1546 |
| 53 | Pre-Exposure Prophylaxis/ | 2274 |
| 54 | (prophylactic* or prophylaxi or prophylaxis).tw,kf. | 164946 |
| 55 | (preventive medication? or preventive therap* or preventive treatment? or protective therap*).tw,kf. | 10949 |
| 56 | (protect* adj3 brain).tw,kf. | 7076 |
| 57 | neuroprotect*.tw,kf. | 64492 |
| 58 | ("no history" adj5 (epileps* or seizure?)).tw,kf. | 271 |
| 59 | or/51-58 [Seizure/Epilepsy prevention] | 248998 |
| 60 | 50 and 59 [Antiseizure Antiepileptic Drugs AND Seizure/Epilepsy prevention] | 8434 |
| 61 | animals/ not humans.sh. | 4682746 |
| 62 | 60 not 61 | 5202 |
| 63 | subarachnoid hemorrhage/ | 20972 |
| 64 | (arachnoidal bleeding or arachnoidal haemorrhage? or arachnoidal hemorrhage? or brain arachnoid haemorrhage? or brain arachnoid hemorrhage? or subarachnoid bleeding or subarachnoid blood or subarachnoid haematoma? or subarachnoid haemorrhage? or subarachnoid hematoma? or subarachnoid hemorrhage? or subarachnoid hemorrhagia or subarachnoidal bleeding or subarachnoidal haemorrhage? or subarachnoidal hemorrhage?).tw,kf. | 26153 |
| 65 | or/63-64 [aneurysmal subarachnoid hemorrhage] | 31251 |
| 66 | 62 and 65 [Antiseizure Antiepileptic AND aneurysmal subarachnoid hemorrhage] | 112 |
| 67 | limit 66 to "all child (0 to 18 years)" | 7 |
| 68 | (adolescent or children).tw. or child, preschool.sh. | 1617577 |
| 69 | infan*.mp. or gestation*.tw. or neonatal.tw. | 1493896 |
| 70 | 66 and (68 or 69) | 2 |
| 71 | 66 not (67 or 70) | 104 |
| 72 | limit 66 to "all adult (19 plus years)" | 53 |
| 73 | adult.mp. or middle aged.sh. or age*.tw. | 9106473 |
| 74 | 66 and 73 | 65 |
| 75 | 71 or 72 or 74 | 112 |
| 76 | limit 75 to english language | 106 |
